# Optimal configurations of spatial scale for grid cell firing under noise and uncertainty

**DOI:** 10.1098/rstb.2013.0290

**Published:** 2014-02-05

**Authors:** Benjamin W. Towse, Caswell Barry, Daniel Bush, Neil Burgess

**Affiliations:** 1 UCL Institute of Behavioural Neuroscience, University College London, London WC1N 3AR, UK; 2 UCL Institute of Neurology, University College London, London WC1N 3AR, UK; 3 UCL Institute of Cognitive Neuroscience, University College London, London WC1N 3AR, UK; 4 UCL Department of Cell and Developmental Biology, University College London, London WC1N 3AR, UK

**Keywords:** grid cell, spatial navigation, Poisson noise, spatial uncertainty

## Abstract

We examined the accuracy with which the location of an agent moving within an environment could be decoded from the simulated firing of systems of grid cells. Grid cells were modelled with Poisson spiking dynamics and organized into multiple ‘modules’ of cells, with firing patterns of similar spatial scale within modules and a wide range of spatial scales across modules. The number of grid cells per module, the spatial scaling factor between modules and the size of the environment were varied. Errors in decoded location can take two forms: small errors of precision and larger errors resulting from ambiguity in decoding periodic firing patterns. With enough cells per module (e.g. eight modules of 100 cells each) grid systems are highly robust to ambiguity errors, even over ranges much larger than the largest grid scale (e.g. over a 500 m range when the maximum grid scale is 264 cm). Results did not depend strongly on the precise organization of scales across modules (geometric, co-prime or random). However, independent spatial noise across modules, which would occur if modules receive independent spatial inputs and might increase with spatial uncertainty, dramatically degrades the performance of the grid system. This effect of spatial uncertainty can be mitigated by uniform expansion of grid scales. Thus, in the realistic regimes simulated here, the optimal overall scale for a grid system represents a trade-off between minimizing spatial uncertainty (requiring large scales) and maximizing precision (requiring small scales). Within this view, the temporary expansion of grid scales observed in novel environments may be an optimal response to increased spatial uncertainty induced by the unfamiliarity of the available spatial cues.

## 1. Introduction

**Figure 1.**
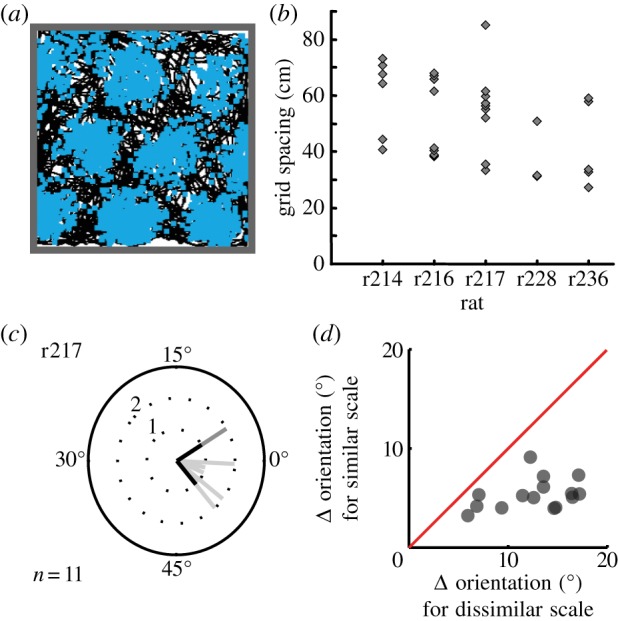
Grid cells exist in modules of discrete scale but similar orientation. (*a*) A grid cell in mEC fires action potentials or ‘spikes’ at the vertices of a triangular grid as the rat forages in a square arena (path shown in black, spikes superimposed), adapted from [2]. (*b*) The grid scales (spacing between neighbouring firing peaks) of the grid cells in each of five rats are clustered around discrete values, adapted from [2]. (*c*) The orientations of the grid-like firing patterns in each module were also significantly clustered, in all five rats. Grid orientations are shown for rat 217 for the small (black), medium (light grey) and large (dark grey) grids, adapted from [2]. (*d*) Differences in the orientations of grids are greater between modules than within modules, but are still significantly clustered between modules (compared with the uniform distribution between 0° and 30° expected for independent modules), adapted from [3] with permission. (Online version in colour.)

Grid cells recorded in the medial entorhinal cortex (mEC) of freely moving rodents fire whenever the animal enters any one of an array of locations arranged at the vertices of a triangular grid across the environment [1]. The spatial scale of the grid-like pattern increases at more ventral recording locations along the mEC [1]. Grid cells are organized into discrete modules such that the cells within each module have firing patterns of similar spatial scale, with a sharp transition in spatial scale between modules [2] (see also [3]). The grid-like firing patterns within a module also have a similar orientation, and grid orientation is clustered (i.e. more similar than expected by chance) across modules within the mEC of a single hemisphere [2,3] (figure 1).

The approximate range of grid scales recorded in rats runs from around 25 cm, i.e. the smallest scale recorded in dorsal mEC, to 500 cm recorded in intermediate/ventral mEC [3,4]. The maximum number of modules recorded within this range is approximately five or six, indicating that there might be 5–10 modules in total [3,4]. It has been suggested that each successive grid scale increases by a fixed factor, producing a geometric series of scales, with scale factors reported in the range between 1.3 and 1.7 [2,3]. However, given the high variability of grid scales within each animal and the difficulty in concurrently observing neighbouring grid modules [3], it is not clear whether this geometric arrangement is precise or just a rough approximation.

**Figure 2.**
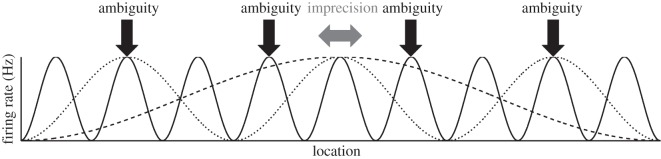
Precision and ambiguity in grid cell firing. The schematic shows the spatial firing patterns of three cells with different spatial scales which fire at their peak rate at the current (central) location of the animal. Horizontal arrow shows uncertainty about the actual position of the animal encoded by the firing of three grid cells with different spatial scales, owing to imprecision in their potentially noisy firing patterns. Vertical arrows indicate potentially ambiguous locations with similar representations in the grid cells’ firing owing to false alignments or near-alignments in firing of subsets of the three cells.

The firing of grid cells within a single module represents the location of the animal within each repeating cell of the grid firing pattern, but does not distinguish between the corresponding locations in different cells of the firing pattern. The *precision* of this representation depends on the level of noise in neural firing, the shape of the spatial distribution of firing and the density of spatial coverage of the grids within the module. Because different modules have different spatial scales, the firing of a population of grid cells containing multiple modules can help to resolve the *ambiguity* of the represented location across corresponding locations within each grid scale, if the ambiguous locations do not align across the modules with different spatial scales (figure 2 and [5–7]). Note that inspection of figure 2 indicates that the difference in scale between adjacent grid scales should be less than a factor of 2, as indicated by the data, so that the smaller scale sinusoid has only one peak within the raised area of the larger sinusoid.

The size of the environment strongly influences the relative importance of the twin problems of ambiguity and precision. In small environments, the problem of ambiguity can be solved by the firing pattern of modules with a scale larger than the environment—these cells do not exhibit repeated firing fields and, as such, are unambiguous. In this case, the accuracy of decoding location from the population firing pattern depends on the spatial distribution of firing (i.e. the density of coverage and the shape of the firing patterns) and the reliability or noisiness of firing, as quantified by Mathis *et al*. [5,6] using mean maximum-likelihood estimate square error.

In large environments, larger than the largest grid scale, the problem of ambiguity dominates (potentially producing much larger errors than those of precision). Maximizing the spatial range over which grid cells could encode location without ambiguity leads to suggestions that the grid scales within an animal should be co-prime, which could theoretically allow representation over a very large range (up to the product of the grid scales [8,9]). However, the effect of noise in neural firing potentially undermines the theoretical capacity of co-prime grid modules, because ambiguity becomes possible between locations where the firing patterns of different modules nearly align (figure 2). The effect of noise is not irrecoverable in small environments [6], but could be much more disruptive in larger environments.

The impact of noisy firing on the spatial range encoded by grid cells and on the optimal organization of grid scales has not been fully explored. Nonetheless, the fact that errors caused by ambiguity will typically be extremely large (i.e. decoded locations will be far from the correct location), leads to the suggestion that combining the grid cell system with a slow-moving representation of location will prove optimal [10]. Such a representation is potentially provided by place cells in the hippocampus [11]. Each place cell typically fires in only a single location [12] so that during normal locomotory activity the overall pattern of activity does not change rapidly. Thus, large errors in the grid cell representation of location could be detected (and potentially corrected) by the fact that they would correspond to sudden large changes in the corresponding place cell representation of location.

Recent experiments have shown that the spatial scale of grid cell firing patterns increases whenever the rat is put into a novel environment [13]. Note that this phenomenon contrasts with the view that grid cells provide a fixed metric for space (e.g. [1]). It also appears to reflect a different mechanism than the parametric response of grid firing patterns to spatial deformation of the environment [2]. When a familiar environment is changed in shape and size, the grids show a partial change in spatial scale, most probably reflecting associations to environmental sensory information, including that mediated by place cells and boundary vector cells [14–16]. By contrast, the expansion of grid firing patterns in a novel environment (which is of the same size as the familiar one) appears to be intrinsically generated.

Currently, it is unknown why this expansion occurs. Here, we suggest that the increase in grid scale is an adaptive response to an increase in spatial uncertainty in a novel environment.

The spatial firing patterns of all grid cells within a single module appear to be coherent, consistent with the presence of attractor dynamics [17,18], which potentially provides a powerful means of reducing the effects of noisy firing by individual cells on the spatial coding of the population. However, the spatial representations of each module can shift relative to each other [17], potentially providing a mechanism for place cell remapping in a new environment. This decoupling of modules implies that spatial uncertainty in a novel environment might cause spatial ‘noise’ reflected in relative movement of different modules, with potentially strong implications for ambiguity errors, even when these shifts are small in amplitude.

To summarize, optimal spatial coding by grid cells will reflect a complex trade-off between precision and ambiguity, which will interact with spatial uncertainty and temporal stochasticity in neural firing and will also interact with the size of the environment relative to the scales of the grids. By simulating the neural firing patterns in a modular grid cell system and estimating decoding error for randomly sampled locations within the environment, we investigate several questions regarding the configurations of grid scale that result in the least decoding error, following [5]. Importantly, we can investigate the impact of noisy firing, environmental size and spatial uncertainty on the decoding error of the simulated grid configurations. The last of these allows us to examine whether the expansion in grid scale observed in novel environments might be an adaptive response that mitigates the effects of spatial uncertainty.

## 2. Material and methods

### (a) One-dimensional grid cell system model

Spiking activity of a population of grid cells, organized into *L* discrete modules by spatial period size, was modelled in a one-dimensional environment using Matlab v. 7 (Mathworks; code may be obtained by contacting the authors). The spike output of a grid cell, *j*, within a particular module, *i*, was modelled, following [5], as a Poisson process with rate modulated by position on an open interval, *x* ∈ (0, *x*_max_), according to a periodic Gaussian tuning curve **α*_i_*_,*j*_(*x*):2.1
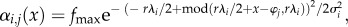
where *f*_max_ is the maximum firing rate (which is constant across the population), **λ*_i_* the baseline spatial period defining the module, *r* the multiplier applied to that spatial period to control grid scale expansion, **φ*_j_* the spatial phase offset, **σ*_i_* the tuning width of the grid fields and mod(*a*,*b*) represents the modulo function.

Within each module with a shared scale **λ*_i_*, tuning curves were created for *M* equidistant spatial phases 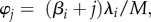 where 0 ≤ *j* < *M* and **β*_i_* is a random additional offset on the interval (0, 1), common to all tuning curves within a module but different between modules. This was added in order to prevent biases that would result from the alignment of tuning curves across modules. Thus, a total of *L*×*M* = *N* neurons were simulated.

### (b) Two-dimensional grid cell system model

The model described in §2*a* was also adapted to model grid cell activity in a two-dimensional environment. Two-dimensional template tuning curves for each grid scale (and expansion thereof) were generated with locations of grid nodes specified as a regular triangular grid with scale *r*λ*_i_* and expected firing rate at each location determined by a Gaussian distribution centred on the nearest node:2.2
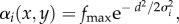
where *d* is the distance from (*x*,*y*) to the nearest grid node.

Within each module, *M* = 195 offset tuning curves were distributed in a 13 × 15 rectangular grid via translations of this original tuning curve, as well as adding a random translation common to all grids in the module. Finally, in a given experiment, all grid tuning curves in all modules were rotated to a common, randomly selected orientation with respect to the environment. All these transformations were performed using cubic interpolation.

### (c) Determining module scales

Three systems for determining relative module scales were used: geometric, co-prime and random. In a geometric system, a set of modules were created by specifying a spatial period multiplier, *p*, a smallest scale (*r* × **λ**_1_) and a total number of modules *L*. The spatial period of each module was determined as *rλ*_*i*_ = *r*λ**_1_*p^i^*^−1^ where 0 ≤ *i* ≤ *L.* In a co-prime system, a set of modules were created with scales in the ratios of prime numbers 2 : 3 : 5 : … (e.g. **λ**_3_ = 5/2**λ**_1_). Finally, random systems were constructed to compare to the geometric system with *p* = 1.4 as follows: 1000 systems were created by taking the smallest and the largest grid scales occurring in the geometric system and selecting a further *L –* 2 scales from a uniform distribution ranging between these scales, hence yielding *L* scales with upper and lower scales matched to the *p* = 1.4 system.

### (d) Modelling spatial uncertainty

Gaussian noise **ɛ*_i_* was generated separately for each module and added to the actual position, *x*, to yield a noisy position estimate *x* + **ɛ*_i_* (in the two-dimensional simulation independent noise was added in both *x-* and *y-*dimensions: *x* + **ɛ*_x_*_,*j*_, *y* + **ɛ*_y_*_,*j*_). The degree of uncertainty was varied by modifying the standard deviation of **ɛ*_i_*. All cells within a module therefore received the same noisy position input, but cells in different modules received different input. Thus, cell firing rate was now modulated according to **α*_i_*_,*j*_(*x* + **ɛ*_i_*). Note that in the two-dimensional simulation, noisy position signals that fell outside the environment were corrected to the closest location at the edge of the environment before being input to the grid cells.

### (e) Decoding

The signal extracted from the grid cell system was the number of spikes, *k*, generated by each neuron during a finite read-out period, *T*—i.e. a population response ***K*** = (*k*_1_,…, *k_N_*)*.* We assume that the decoding cannot take the added noise into account in any way, so that given a position *x* the probability of observing the response ***K*** in time *T*, following [5], is taken to be:2.3
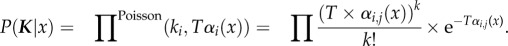


From the population response ***K***, we can decode position as the maximum-likelihood estimate of *x*, that is 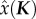. Given the initial assumption that all values of *x* within the environment are uniformly likely2.4
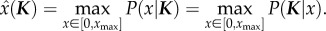


Thus, 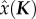 may be closely approximated by calculating *P*(***K****|x*) for a sufficiently finely spaced uniform sample of *x* values on the interval [0,*x*_max_], and selecting the value of *x* which yields the greatest *P*(***K****|x*). We used a spatial bin size of **Δ*x* = 0.5 cm. Where two or more values of *x* yielded the same maximal *P*(***K****|x*) (i.e. decoding was ambiguous), one was randomly selected.

In two dimensions, **α*_i_*_,*j*_(*x*,*y*) is calculated by cubic interpolation from the tuning curve, which gives expected firing rates only at the sampled intervals. As all possible locations are considered independently in the probability calculations, no further adaptation is required to implement this in two dimensions.

### (f) Measuring error

The mean maximum-likelihood estimate square error, or MMLE, assesses the accuracy of decoding possible with a particular grid system, based on the square error of position decoding. Exact MMLE is defined [19,20] as2.5
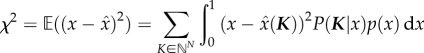
and in two dimensions2.6
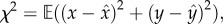
where 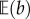 is the expected value of a random variable *b*. MMLE values for each set of grid cell network parameters were estimated using the Monte Carlo method. For each iteration *c*, a sample position *x_c_* was selected, and with the introduction of noise **ɛ** to the modelled grid cells, a population spike response *K_c_* was generated, then decoded to yield 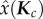. With a large number of iterations, MMLE can be approximated [5]. One thousand iterations were performed (i.e. 1 ≤ *c* ≤ 1000)2.7
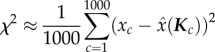
and in two dimensions2.8
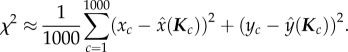
For a given set of parameters in a geometric or co-prime grid system, 10 such experiments were performed to calculate 10 independent estimates of MMLE, unless specified. For the random grid scales, a single 1000 iteration experiment was performed for each of the 1000 generated systems.

### (g) Comparison of decoding performance to chance levels

For the purposes of comparison, chance performance levels were calculated for each track size (i.e. corresponding to a uniform distribution of decoded locations). For a one-dimensional environment, this was *d*^2^/6 and for a square two-dimensional environment *d*^2^/3.

### (h) Parameters

In all simulations, the following parameters were used, following Mathis *et al*. [5]: read-out time period, *T* = 0.1 s, the approximate length of a theta cycle; maximal grid firing modulation rate *f*_max_ = 10 Hz; smallest baseline spatial period, **λ**_1_ = 25 cm; total number of modules, *L* = 8; tuning width of grid pattern bumps:2.8
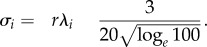


In one dimension, the number of equidistant phase offsets represented within a module of grid cells, *M* = 20 or *M* = 100 and *M* = 195 in two dimensions; thus the total number of cells, *N* = *L* × *M* = 160 or 800 in one dimension and *N* = 1560 in two dimensions.

## 3. Results

How does the arrangement of the spatial scales of grid cell modules affect spatial encoding when the stochastic nature of neural firing is taken into account? We simulated systems of Poisson firing grid cells containing eight modules of 20 or 100 cells per module, with geometric, co-prime and random series of grid scales, all starting from the smallest module with scale of 25 cm. First, we examined the effect that the configuration of spatial scales, the number of cells per module and the size of the environment have on the performance of the grid system in terms of the decoding error. Then, we consider the optimal response of a grid system to independent variability in the estimated location across modules, which might correspond to the effect of spatial uncertainty in a novel environment.

### (a) Configuration of spatial scales across modules

**Figure 3.**
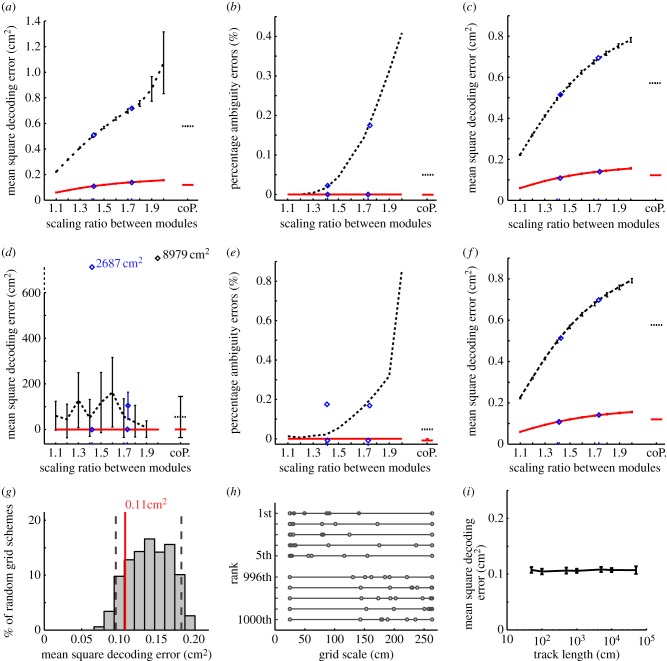
Decoding error in grid systems as a function of the configuration of grid scales across modules. (*a*) Mean squared decoding error on a 1 m track for geometric grid systems with scale ratios 1.1–2.0 as well as √2 (1.41) and √3 (1.73) (diamonds). Errors for a co-prime grid system are presented on the far right. Results are shown for systems with 20 (dashed line) and 100 grid cells per module (solid line). Error bars indicate the s.e.m. of 1000 simulations each consisting of 1000 decodings of random locations from grid cell activity. (*b*) Percentage of decodings on the 1 m track resulting in large errors (error > 10 cm^2^) attributed to decoding ambiguity. (*c*) Decoding errors (as in *a*) with the large (ambiguity) errors removed. (*d–f*) Simulations on 18 m track. (*d*) Mean squared decoding errors (the points for ratio√2 and 2.0 are off the scale). (*e*) Percentage ambiguity errors. (*f*) Decoding error with large (ambiguity) errors removed. (*g*) Decoding errors for 1000 grid systems with random scales matched to a geometric system with scale factor 1.4 (18 m track, 100 cells per module; grey bars). The 5th and 95th percentile of the random population are shown as grey dashed lines—the matched geometric system lies at the 15.4th percentile (solid vertical line). (*h*) The actual spatial scales of the modules in the best and worst performing random systems. (*i*) Decoding error for a geometric system (factor 1.4, 100 cells per module) in one-dimensional environments of increasing size (50 cm–500 m). (Online version in colour.)

Firstly, we examined scaling factors between adjacent grid scales ranging from 1.1 to 2.0, including factors √2 (1.41) and √3 (1.73), and estimated the decoding error of each grid cell system on a 1 m linear track (see §2*e*,*f*). Figure 3*a* shows that decoding error is very low overall (squared error generally being less than 1 cm^2^), with an improvement in encoding accuracy for 100 cells per module compared with 20 cells per module—presumably because Poisson firing noise is averaged across a larger cell population. In addition, there is a moderate effect of the geometric ratio on encoding accuracy, such that the smaller ratios, which have more small-scale grids, are more accurate. The performance of the system with ratio 2 and 20 cells per module is particularly poor, with a high variance indicating the presence of two types of error (reflecting precision and ambiguity, respectively).

The presence of two types of error is illustrated by figure 3*b*, where the incidence of large amplitude errors (i.e. instances of decoding with squared error greater than 10 cm^2^) generated by different scaling factors is quantified. These errors are larger than would be expected if they were owing to imprecision in the smallest grid scale, and probably reflect decoding ambiguity. Figure 3*b* shows that these ambiguity errors begin to appear in grid systems with 20 cells per module as the scale ratio approaches 2. Although these occur very infrequently (in less than 0.5% of decoding trials), the errors are large and contribute disproportionately to the mean squared error. For example, in the 1.9 ratio system with 20 cells per module, 0.31% of trials produced large errors and these have a mean square size of 38.1 cm^2^, whereas the remaining trials have a mean squared error of 0.75 cm^2^. In the grid systems with 100 cells per module, no ambiguity errors occurred in any of the 10^6^ simulated trials. Figure 3*c* replots the data from figure 3*a* with the ambiguity errors removed—as expected, the performance of the 20 cells per module system is improved at larger geometric ratios and the variability is reduced.

The presence of ambiguity errors in grid systems with 20 cells per module is clearer in 18 m linear environments (figure 3*d*). The mean decoding error is dominated by the infrequent but very large decoding ambiguity errors, which also cause large variance. Note the decoding accuracy allowed by geometric ratio 2, and to a lesser extent √2, is particularly poor and exceeds the limits of the *y*-axis (mean squared decoding error 8979 and 2687 cm^2^, respectively). The proportion of trials showing decoding ambiguity errors is shown in figure 3*e*. These errors occur for all grid systems with 20 cells per module and their amplitudes are increased relative to the 1 m track (because the 18 m track provides greater scope for larger errors). Taking the 1.9 scale ratio, again these errors account for 0.32% of the trials, but their mean square size was 2500 cm^2^, while the size of the errors in the remaining trials was effectively unchanged at 0.76 cm^2^. The geometric ratio 2 coding scheme in particular suffers from a large number of decoding ambiguity errors on 0.86% of trials, indicating the inefficacy of integer scale ratios (figure 2). The geometric ratio √2 scheme also exhibits a disproportionate number of ambiguity errors when compared with the similarly scaled 1.4 and 1.5 schemes—this appears to reflect the fact that under the √2 scheme alternate grid modules follow a geometric progression with ratio 2. Again the grid system with 100 cells per module does not generate ambiguity errors. Decoding error for the same grid systems is shown without the infrequent, but very large, decoding ambiguity errors in figure 3*f*. As with figure 3*c*, this shows that the remaining (precision) errors increase with increasing scale ratio, as would be expected from the concomitant increases in the breadths of tuning of the grid firing fields in all but the smallest scale module.

The performance of configurations of grid modules with a co-prime sequence of scales (i.e. a 2 : 3 : 5 : 7 : 11 : 13 : 17 : 19 ratio of scales, starting from 25 cm and ending at 237.5 cm) is similar to a geometric series with scale factor approximately 1.5 (range 25–427 cm) for both the 1 and 18 m tracks, and in systems with 20 and 100 cells per module (see the rightmost points in figure 3*a–f*). It performs slightly worse than the geometric series with scale factor 1.4, whose overall range (25–264 cm) is best matched to it. Thus, there seems to be no specific advantage for a co-prime series of grid scales over a geometric series in one-dimensional environments of these sizes. Grid systems with geometric ratio 1, i.e. where all grids are 25 cm in scale, were also simulated, but the data are not shown because they give such large errors, being unable to disambiguate locations more than 25 cm apart (e.g. mean squared error with 20 cells per module on a 1 m track is 1669 cm^2^).

Figure 3*g* examines the decoding error for grid systems with a random distribution of grid scales between 25 and 264 cm, for comparison with a geometric series with scale ratio 1.4 (which has the same range of scales and is investigated further below). The mean error in the geometric system lies at the 15.4th percentile of the distribution of random scales, showing that on average a geometric series performs somewhat better than a random series with a similar range of scales but that this advantage is slight and all systems exhibit only precision errors. The five randomly generated grid systems that gave the lowest decoding errors (rank first to fifth) as well as the five yielding the highest errors (rank 996th to 1000th) are shown in figure 3*h*. The best performing random systems include more small-scale grid modules than the poorly performing systems, which are dominated by larger scale grids, and so somewhat resemble the geometric series of scales. This reflects the fact that, on the 18 m track with 100 grids per module, ambiguity errors are unlikely to occur, and so the maximum decoding accuracy is obtained by minimizing precision errors—hence small grid scales are favoured.

Figure 3*i* provides an indication of the actual capacity of the grid system and how this compares to the 18 m track used in the previous simulations. Specifically, decoding error of a geometric system with scale ratio 1.4 and 100 grids per module is examined on tracks of increasing length. In all cases, the decoding errors are small, consisting mainly of precision errors even on the largest track (500 m), suggesting that the maximum range of this system is considerably larger than this value, as suggested by Fiete *et al*. [8].

These initial simulations demonstrate several points. The presence of two types of error is clearly shown: precision errors which are common but relatively small in magnitude and ambiguity errors which are infrequent but potentially very large. The small decoding errors resulting from precision errors are reduced further in grid systems with more small-scale grid modules and also in systems with more cells per module. Although ambiguity errors are infrequent, typically occurring in less than 1% of decodes, their large size was shown to disproportionally degrade the system's performance. Ambiguity errors were found to be more prevalent in systems with fewer cells per module (20 versus 100) as well as in the larger environment (18 versus 1 m) where their magnitude was also increased. We did not see any specific advantage for the co-prime system over a similarly scaled geometric system. However, the geometric system following a ratio of 2 between modules performed poorly owing to a disproportionate number of ambiguity errors on the 18 m track—to a lesser extent this was also true for the ratio √2 system.

### (b) Optimal response to independent spatial uncertainty across modules

Given our conclusions in §3*a*, that a geometric series of grid scales across modules performs as well as any other configuration, we chose to use a geometric series with a scaling ratio of 1.4 for the remaining analyses. Such a scaling ratio is indicated (on average) by the data in [3], although we note that a larger ratio would be required to produce a range comparable with the smallest and the largest grid scales that have been reported (i.e. 25–500 cm [4]) with only eight modules, more consistent with the ratio in [2]. We used 100 cells per module, because this minimizes the effect of decoding ambiguity errors arising from Poisson firing. Finally, we decode position on an 18 m track, rather than a 1 m track, because this is closer to the natural situation in which grid systems must operate, where the range of the animal is larger than the largest grid scale, and in which the combinatorial power of the grid code can be exploited.

As noted in §1, the spatial firing patterns of the grid cells within the same module appear to be coherent [18], and we have seen that increasing the number of grid cells within each module mitigates the effects of noisy firing (§3*a*). Thus, the encoding of location within each module appears to be robust. However, the spatial representations of each module can shift relative to each other [3]. This decoupling implies that each module performs its own independent estimation of location (e.g. each receiving independent movement and/or location-related signals). This type of spatial noise or uncertainty will cause shifts in the relative locations represented by different modules, with potentially strong implications for ambiguity errors. Following the experimental observation of grid scale expansion in novel environments [13], we examined whether a uniform expansion of all grid scales might be an optimal response to spatial uncertainty in terms of reducing the decoding error.

Spatial uncertainty was simulated by adding random offsets in the locations represented by different grid modules, and all grid scales were multiplied by a single expansion factor. The offsets were taken from Gaussian distributions with zero mean, increasing the standard deviation to simulate increasing uncertainty (see §2*d* for details).

**Figure 4.**
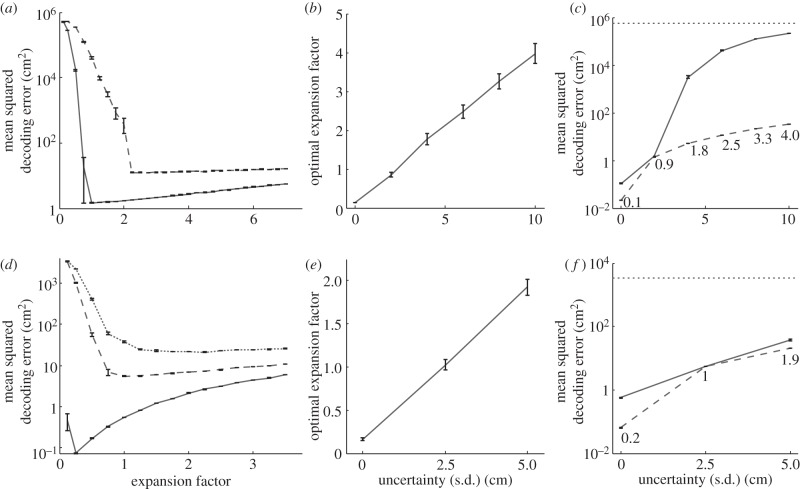
Expansion of a grid system is an optimal response to spatial uncertainty. (*a*) Mean squared decoding error on an 18 m track in grid systems subjected to varying expansion, under low uncertainty (s.d. 2 cm; solid line) and higher uncertainty (s.d. 6 cm; dashed line). For each level of uncertainty, there is an optimal expansion factor that minimizes decoding error, and grid scale expansions smaller or larger than this will result in greater errors. (*b*) The mean optimal expansion factor is greater for higher levels of uncertainty; this relationship appears linear. (*c*) Mean squared decoding error for baseline (solid line) and optimally expanded (dashed line) grid systems, and for performance at chance (dotted line). Labels indicate mean optimal expansion. (*d*), (*e*) and (*f*) show simulations in a two-dimensional 1 m^2^ environment and are equivalent to (*a*), (*b*) and (*c*), respectively. Error bars are the s.e.m. of 10 runs of the simulation per set of conditions, each consisting of 1000 decodes of random locations.

Figure 4*a* shows the decoding error for the grid system, for two levels of uncertainty, as a function of the grid scale expansion factor in the range 0.125–7. For both levels of uncertainty, small expansion factors lead to large decoding errors, reflecting the occurrence of decoding ambiguity errors caused by spatial uncertainty (for this grid system and environment, Poisson firing alone does not cause ambiguity errors, or at least does so only extremely rarely, owing to population coding; see §3*a*). Equally, for larger expansion factors, the overall decoding error increases owing to decreasing precision. The scaling factor representing the optimal trade-off between these two factors depends on the level of uncertainty. In fact, the optimal expansion factor, which minimizes decoding error in this situation, increases linearly with the level of spatial uncertainty, as illustrated in figure 4*b*. The differences in decoding error generated by the optimally expanded and initial (unexpanded) grid systems are shown in figure 4*c*.

A similar pattern of results is generated by the grid system in a two-dimensional, 1 m^2^ environment (see figure 4*d–f*, where expansion factors ranging from 0.125 to 3.5 were assessed). Note that at low uncertainty (s.d. 2.5 cm) the optimal grid expansion is 1.0 (i.e. comparable to ‘baseline’ scales measured empirically), and with an increase in uncertainty (to 5 cm) the optimal expansion is 1.9, which is of a similar order of magnitude to the expansion recorded empirically in exposure to a novel environment [13].

At zero spatial uncertainty, the optimal expansion factor is less than unity and represents shrinkage of the grid firing pattern. In fact, the true optimum is likely to be even smaller than that observed here. Our estimate was limited by the range examined, and expansion factors smaller than 0.125 were not examined. However, the fact that these shrunken grids can code location with so little error is further evidence of the power of grid systems to encode unique locations over ranges much larger than their scales.

In summary, using simulations of one- and two-dimensional environments, we have modelled uncertainty as independent spatial noise affecting each grid module. Elevated spatial uncertainty was shown to greatly increase the occurrence of ambiguity errors, resulting in a pronounced reduction in spatial coding accuracy. However, the deleterious effect of spatial uncertainty is less pronounced in systems with larger scale grids. Hence, a uniform expansion of all grid modules in a system was seen to mitigate the effect of spatial uncertainty, reducing the decoding errors produced by ambiguity errors.

We interpret our results as suggesting that the grid expansion observed *in vivo* [2] could be an optimal response to spatial uncertainty, which is assumed to produce independent spatial error in the locations represented by different grid modules. Expanding grid scales appears to reduce the effect of uncertainty, and optimal expansion represents a trade-off between mitigating uncertainty and maintaining sufficient precision. The optimal expansion factor is linearly proportional to the uncertainty. The likely explanation is that grid expansion is required to keep the size of the jitter small enough relative to the sizes of the grids to avoid ambiguity errors, but not to expand more than necessary (as this will reduce precision). The optimal expansion appears to approximately maintain the uncertainty (i.e. the standard deviation of the distribution of spatial error) below 10% of the smallest grid scale (i.e. 2.5 cm jitter with 25 cm smallest grid scale; 5 cm jitter with 50 cm smallest grid scale, etc.).

## 4. Discussion

Using maximum-likelihood decoding of simulated grid cell Poisson spiking dynamics, we examined the accuracy with which populations of grid cells can encode position in one- and two-dimensional environments. We explored the effects of varying the number of grid cells per module, the scaling factor between modules and the environment size. Additionally, we described the effect of spatial uncertainty—modelled as independent noise in the relative position of different grid modules—on encoding accuracy as well as the interaction between spatial uncertainty and grid scale. In particular, we showed that increased spatial uncertainty markedly reduces the precision with which location is encoded. Finally, we demonstrate that this reduction in performance can be minimized by a homogeneous expansion of all grid scales.

Our simulations demonstrate that grid systems are susceptible to two types of error: localized precision errors which reflect small inaccuracies in the decoded location and larger ambiguity errors resulting from decoding location to the wrong part of the track. In the absence of spatial uncertainty, the sole source of noise in the system arises from the Poisson dynamics, and ambiguity errors occur relatively infrequently. However, their magnitude scales with the size of the environment, unlike precision errors. As such, even in a moderately sized environment, ambiguity introduces significant errors that are typically of the orders of magnitude larger than the precision errors (which are usually much smaller than a rodent's body length). Grid systems with smaller grid scales experienced smaller precision errors and so, in the absence of ambiguity errors, were more accurate. This effect was also reflected in the performance of grid systems with randomly selected scales—those providing the most precise information about location included more small-scale grid modules.

Increasing the number of grid cells per module also decreased the size of the precision errors. This effect appears to arise both because Poisson noise is averaged across cells and because the larger population of cells provides a better approximation to the idealized population vector for a given position. However, increasing the number of cells per module also decreased the incidence of ambiguity errors. Partly this effect occurs because averaging the Poisson noise across more cells renders it less likely that the grid code for two different locations will be confused. As such, the number of different states that the grid code can disambiguate is increased—this enhances both the precision and range of the code. Indeed, with 100 cells per module the range of the grid code is combinatorially huge (greater than 500 m), easily exceeding the scale of the largest grid module (264 cm, given a geometric ratio of 1.4).

Previous theoretical work has suggested that the scale of grid modules should follow a co-prime sequence [8]. In our simulations, grid systems based on co-prime and non-integer geometric scaling factors perform equally well, notwithstanding the slight reduction in the magnitude of precision errors for the grid systems with smaller scale modules. However, systems based on a geometric scaling factor of 1 and 2 perform particularly poorly, generating more ambiguity errors. In the former, this is because all modules contain grids of the same scale, making it impossible to disambiguate positions further apart than a single grid spatial period (25 cm). The weakness of the factor 2 system appears to arise because all modules’ scales share a common integer factor which increases the likelihood of ambiguity errors, as discussed by Fiete *et al*. [8]. Interestingly, the weakness of the factor 2 code is most apparent on longer tracks (18 versus 1 m), suggesting that simulations of even larger environments might potentially reveal further differences between co-prime and geometric systems.

Finally, we also showed that uniform expansion of grid scale is an optimal response to spatial uncertainty—larger scales provide more accurate representations of position in high uncertainty situations. Again, this effect can be understood in terms of precision and ambiguity errors. With low spatial uncertainty, ambiguity errors are unlikely, and uniformly increasing grid scale simply increases the size of the precision errors—thus smaller grid scales are favoured. With increasing spatial uncertainty, ambiguity errors occur more frequently, but this can be mitigated by increasing grid scales. There appear to be two reasons for this. Firstly, increasing scale reduces the size of spatial uncertainty relative to the grids, decreasing the chance of making ambiguity errors. Secondly, it increases the range of the grid system, such that a given environment occupies less of the overall capacity. Because location is only decoded to locations within the environment, this effectively means fewer candidate decode locations are considered, again reducing the chance of ambiguity errors. This form of ‘capacity’-based error correction was previously described in the context of a non-expanded grid system by Fiete *et al*. [8].

To conclude, increasing spatial uncertainty reduces the fidelity and range of the grid system, and these effects can be mitigated by uniform expansion of grid scale. This provides a potential explanation for the transient expansion of grid scales observed when an animal is placed in a novel environment [13]. We suggest that novel environments are characterized by increased spatial uncertainty, the animal being unfamiliar with the form and reliability of the available spatial cues. In such circumstances, to minimize degradation of the spatial encoding, the grid system expands. As the environment becomes more familiar and the animal learns about the available cues, spatial uncertainty reduces, and the grid scale returns to baseline levels. It may be that expansion is mediated by changes in the theta-band oscillatory dynamics of grid cell firing that co-occurs with the expansion [13], as would be consistent with models in which these oscillatory dynamics determine the spatial firing pattern [21–23]. In turn, it seems possible that the changes in theta-band dynamics may be triggered by increased levels of acetylcholine in the hippocampal formation: elevated acetylcholine tone is associated with environmental novelty [24], is known to modulate the oscillatory dynamics of mEC stellate cells [25] and has been theoretically implicated in signalling uncertainty [26]. Additionally, grid expansion occurring in response to increased spatial uncertainty might promote the ‘remapping’ of place cell firing, which also co-occurs with the expansion [13,27]. This would be consistent with models suggesting that remapping reflects a mismatch between path integration-based grid inputs and environmental sensory inputs to place cells [13,28].

## Funding statement

This work was funded by a Wellcome Trust Principal Research Fellowship to N.B., a Wellcome Trust UCL 4 Year PhD Neuroscience Studentship to B.W.T. and a Sir Henry Dale Fellowship jointly funded by the Wellcome Trust and Royal Society to C.B. Some exploratory work was carried out by B.W.T. during the Okinawa Computational Neuroscience Course 2012 at the Okinawa Institute of Science & Technology.
